# Time-Series Representation Feature Refinement with a Learnable Masking Augmentation Framework in Contrastive Learning

**DOI:** 10.3390/s24247932

**Published:** 2024-12-11

**Authors:** Junyeop Lee, Insung Ham, Yongmin Kim, Hanseok Ko

**Affiliations:** School of Electrical Engineering, Korea University, Seoul 02841, Republic of Korea; junyeoplee94@gmail.com (J.L.); billado@korea.ac.kr (I.H.); dydals3819@korea.ac.kr (Y.K.)

**Keywords:** time-series representation, contrastive learning, masking augmentation

## Abstract

In this study, we propose a novel framework for time-series representation learning that integrates a learnable masking-augmentation strategy into a contrastive learning framework. Time-series data pose challenges due to their temporal dependencies and feature-extraction complexities. To address these challenges, we introduce a masking-based reconstruction approach within a contrastive learning context, aiming to enhance the model’s ability to learn discriminative temporal features. Our method leverages self-supervised learning to effectively capture both global and local patterns by strategically masking segments of the time-series data and reconstructing them, which aids in revealing nuanced temporal dependencies. We utilize learnable masking as a dynamic augmentation technique, which enables the model to optimize contextual relationships in the data and extract meaningful representations that are both context-aware and robust. Extensive experiments were conducted on multiple time-series datasets, including SleepEDF-78, 20, UCI-HAR, achieving improvements of 2%, 2.55%, and 3.89% each and similar performance on Epilepsy in accuracy over baseline methods. Our results show significant performance gains compared to existing methods, highlighting the potential of our framework to advance the field of time-series analysis by improving the quality of learned representations and enhancing downstream task performance.

## 1. Introduction

**Figure 1:**
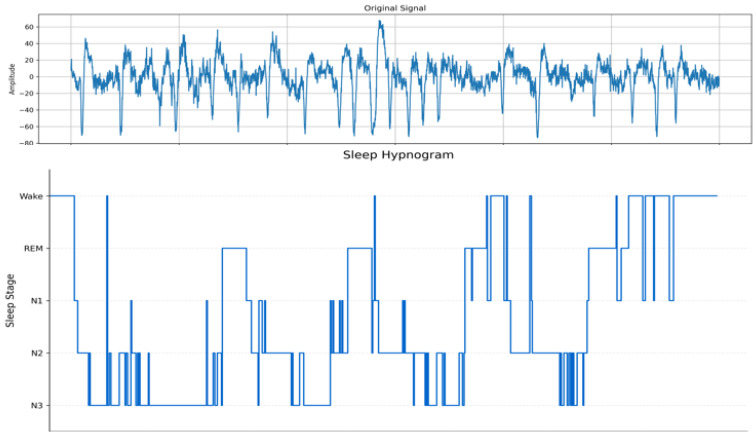
The structure and example of the time series data of the Sleep-EDF dataset. The dataset above represents an example obtained from the Sleep-EDF collection, where the waveform illustrates the time-series data, and the annotations below depict the corresponding time-series labels.

Time-series data are widely utilized across various fields, including medical sleep analysis, dynamic systems, and behavioral analysis [1,2,3,4,5,6]. Learning effective representations and conducting a comprehensive analysis of this data are paramount steps toward developing improved systems in these areas. However, time-series data often consist of a large volume of signals, sometimes spanning multiple channels, making it challenging for humans to interpret and analyze directly [7,8,9,10,11]. Current methods often struggle to effectively balance global temporal dependencies and local feature extraction, which this study addresses through a novel integration of masking and contrastive learning. Furthermore, a significant difference between time-series data and the more extensively studied image data is the continuity inherent in time-series data [12]. This continuity increases complexity, reducing interpretability and presenting additional challenges in terms of labeling and annotation [13]. Figure 1 highlights the intrinsic characteristics of time-series data that make it difficult for direct human interpretation and analysis. The sequential nature, intricate dependencies, and high-dimensional features in time-series data create significant challenges for manual understanding [14,15]. Moreover, time-series data exhibit not only continuity but also enhanced correlations across the entire labeled dataset, making it essential to identify subtle elements and apply direct data augmentation.

Numerous deep-learning methods have been proposed to address these challenges. However, unlike image datasets, the relatively smaller amount of labeled time-series data necessitates a deeper understanding of their unique characteristics and features [16].

Self-supervised learning has garnered significant attention recently, as it enables effective representation learning from unlabeled datasets. Compared to fully supervised models, self-supervised models have demonstrated strong performance, proving effective in tasks such as image classification and segmentation using various approaches [17,18]. Self-supervised learning is an approach that identifies discriminative features by generating pseudo-labeled intermediate representations within unlabeled datasets. This technique enables models to learn useful representations without extensive labeled data, allowing for more effective feature extraction and enhancing performance in downstream tasks [19].

Contrastive representation learning, recognized as a powerful method in self-supervised learning, operates by generating positive and negative pairs through data augmentation. By leveraging pre-training tasks, in a nutshell, it encodes representations that enable the model to learn shared features within the latent space, aligning common characteristics across different perspectives. This approach effectively captures nuanced patterns and relationships within the data, enhancing the model’s capacity to generalize in downstream tasks [20]. Notably, contrastive learning offers a promising solution for time-series data, where extracting discriminative features often poses a significant challenge. This approach capitalizes on diverse, augmented datasets to derive distinctive and meaningful representations, allowing the model to capture subtle patterns that might otherwise be overlooked. By learning to differentiate between augmented data variations, contrastive learning enhances the model’s ability to recognize essential features, making it particularly suited for complex time-series analysis [21].

Augmentation is a fundamental process to contrastive learning, functioning as a critical pre-training mechanism that exposes the model to diverse perspectives of the same dataset. By systematically altering data views, augmentation enables the model to learn to bring positive pairs closer together in feature space while pushing negative pairs farther apart. This strategic manipulation enhances the model’s capacity to capture subtle yet discriminative features, cultivating representations that are not only well separated but also robust to data variability. Such refined representations are particularly advantageous for downstream tasks, as they enable the model to generalize effectively and maintain accuracy even in complex, real-world applications where feature separability is crucial [22].

Despite recent advancements, applying augmentation techniques to time-series data continues to demand substantial research. Many existing augmentation methods have been developed with a primary focus on image data, often failing to account for the temporal and sequential nature of time-series data [23,24,25]. As a result, these techniques lack the specificity and adaptability required for effective time-series augmentation. Additionally, they have not undergone a systematic or comprehensive review tailored to time-series applications, underscoring the need for the rigorous exploration of augmentation strategies that can capture the unique patterns and complexities inherent in time-series data [26].

The masking reconstruction technique represents a favorable approach to feature extraction, designed to capture both global contexts and local nuances by training a neural network to reconstruct deliberately masked portions of data. Through this process, the network learns to anticipate and fill in missing segments, enabling it to understand intricate dependencies within the data structure. This capability is beneficial for time-series data, where both spatial coherence and temporal continuity are essential for meaningful representation. By reconstructing masked regions, the network gains insights into subtle temporal patterns and spatial relationships, allowing it to identify and model sequential dependencies. Consequently, this method is suited to time-series data applications, as it effectively balances the need for comprehensive, high-level pattern recognition with the ability to discern fine-grained, temporally ordered details. This dual-focus approach supports robust model training, enhancing the network’s adaptability and accuracy across complex, real-world time-series tasks.

**Figure 2:**
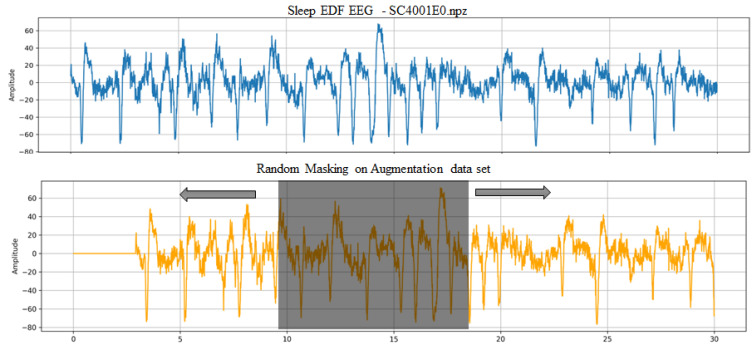
Examples of the time-augmented data with masking employed. We employ a reconstruction model that facilitates the reconstitution of masked segments within the data, allowing us to extract a more comprehensive understanding of both local and global structural elements.

Figure 2 provides illustrative examples of this study’s time-augmentation and masking augmentation techniques. These examples demonstrate augmentations that were applied to the original time-series data to enhance the model’s ability to learn robust and discriminative features. When adapting conventional augmentation methods, a frequent struggle is in effectively capturing the inherent continuity and subtle patterns of time-series data, particularly in complex datasets like Sleep-EDF. In that regard, adopting a fusion strategy with time augmentation, which introduces variability in temporal patterns, and masking augmentation, which selectively obscures parts of the signal, allows the model to focus on reconstructing and understanding underlying structures. Specifically, by employing masking to enhance feature extraction in a contrastive learning framework, we aim to explore how this combined method can effectively capture temporal continuity and discriminative features within time-series data.

To the best of our knowledge, this study represents the first attempt to integrate masking techniques with a contrastive learning approach specifically for time-series data representation. This novel integration has the potential to advance time-series analysis by providing more robust, context-aware representations that align with the unique demands of sequential data.

Inspired by these considerations, we present a novel contrastive learning framework that integrates learnable masking augmentation tailored for time representation learning. This innovative approach aims to enhance the model’s capacity to discern complex temporal patterns while simultaneously improving the discrimination of salient features within time-series data. By leveraging learnable masking, the framework dynamically adjusts the masking process to optimize the learning of contextual relationships and dependencies, thereby facilitating a more nuanced understanding of the underlying data structure. Ultimately, this method aspires to push the boundaries of time-series analysis by providing a robust mechanism for capturing both the intricacies of temporal dynamics and the essential features necessary for accurate representation.

In pursuit of this objective, our focus is centered on the effective reconstruction of augmented data as a pre-training task, which facilitates learning diverse features. By applying these learned features within a contrastive learning framework, we aim to enhance the effectiveness of time representation learning significantly. This methodology not only strives to refine feature extraction but also seeks to optimize the overall learning process, allowing for a more profound comprehension of the intricate dynamics inherent in time-series data. Through this approach, we endeavor to bridge the gap between raw data and meaningful representations, ultimately contributing to advancements in time-series analysis and modeling. The contribution of this work is summarized as follows:

- It introduces a comprehensive framework for contrastive learning through the integration of a learnable masking network with a conventional contrastive learning framework, culminating in the establishment of a loss function. This innovative process not only incorporates existing augmentation techniques but also enhances them by enabling the model to learn and refine features more effectively. By focusing on feature learning in conjunction with the masking mechanism, we aspire to achieve superior feature refinement, ultimately yielding enhanced augmentation features that improve the model’s performance in time representation tasks. This integrated approach has the potential to advance the field of time-series analysis by providing more robust and context-aware representations.
- It proposes a feature extraction module that harnesses intermediate features acquired during the reconstruction of masked time-series data to significantly enhance feature utilization in contrastive learning. By emphasizing the importance of these intermediate representations, we aim to optimize the model’s ability to extract meaningful features that encapsulate the complexities inherent in time-series data. This process not only improves the overall effectiveness of contrastive learning but also facilitates a more nuanced understanding of temporal dynamics, ultimately contributing to advancements in time representation tasks.
- It validates the effectiveness of the proposed approach to time representation learning by conducting performance evaluations across a diverse range of datasets, including the HAR dataset [27], Sleep-EDF [28], and Epilepsy [29] datasets. This comprehensive analysis aims to demonstrate the robustness and applicability of our method in various contexts.

This study is structured as follows: Abstract, Introduction, Related Work, Methodology, Experiment, Discussion, and Conclusions. The Abstract provides an overall summary, encapsulating the study’s objectives, methods, and findings. Section 1 presents the study’s motivation and background, establishing the importance of the research and highlighting key challenges. In Section 2, we review relative research and contextualize the study by discussing foundational methods and advancements in the research field. Section 3 details the specific techniques and frameworks applied in our approach, offering a particular description of the modules and losses utilized to achieve our objectives. Section 4 showcases the performance results of the proposed model, along with an in-depth analysis of its effectiveness across various datasets. Finally, Section 5 consolidates the key insights gained and discusses implications and potential avenues for future research.

## 2. Related Work

Time representation learning has matured through diverse approaches and frameworks, each bringing distinct contributions to how temporal data can be effectively modeled and understood. In this section, we undertake a systematic review of self-supervised learning, beginning with foundational studies that pioneered this approach, followed by its specialized applications in time-series contexts, where the challenge of limited labeled data makes self-supervised techniques invaluable. Through self-supervised learning, models can leverage intrinsic temporal structures without requiring extensive manual labeling, making it especially relevant for time-series data with continuous, often unlabeled, sequences.

Following this, we examine contrastive learning, a technique that has emerged as a robust solution for extracting discriminative features in time-series representation learning. By creating positive and negative sample pairs through data augmentations, contrastive learning enables models to distinguish fine-grained temporal patterns, a process essential for capturing the complex, sequential nature of time-series data. We focus on how contrastive learning has been explicitly tailored for time-series applications, addressing challenges unique to this domain, such as handling variable sequence lengths, ensuring temporal coherence, and managing high dimensionality.

Additionally, we explore the augmentation strategies central to contrastive learning, which are designed to expand the data’s representational richness by generating multiple variations of each time sequence. Although well-studied in computer vision, such augmentations require unique adaptations for time-series data to ensure that essential temporal dependencies are maintained. To build on these augmentations, we propose integrating a reconstruction-based approach, which aims to enhance feature granularity by learning to reconstruct masked or distorted segments of the time-series data. This method not only reinforces the temporal coherence of representations but also provides additional avenues for capturing subtle patterns within sequences.

Together, these approaches—self-supervised learning, contrastive learning, augmentation, and reconstruction—serve as the foundation of our framework, enhancing the depth and robustness of time-series representation learning. Additionally, we introduce methods to enhance the extracted features, strengthening their robustness and relevance for downstream tasks. Consequently, a comprehensive review of related research is explored in detail in the following sections, laying the groundwork for our proposed methodology.

### 2.1. Self-Supervised Learning in Time Series Representation

Self-supervised learning has emerged as a transformative paradigm within the broader category of unsupervised learning, distinguished by its ability to extract supervisory signals from unlabeled data. By leveraging inherent structures and relationships within the data, self-supervised learning enables the generation of rich, meaningful representations that can be effectively applied across a spectrum of downstream tasks, including classification, regression, and clustering. This innovative approach mitigates the reliance on extensive labeled datasets, addressing a significant bottleneck in many machine learning applications.

Self-supervised learning methodologies in time series representation can be categorized into three principal frameworks, generative-based methods, contrastive-based methods, and adversarial methods, each offering unique advantages and operational mechanics [19].

Generative-based methods focus on data synthesis for tasks such as forecasting and reconstruction. These methods excel in tasks such as forecasting and reconstruction, where the goal is to predict future data points or reconstruct original data from noisy inputs. Techniques like auto-regressive modeling estimate the data distribution by predicting each data point sequentially based on its predecessors, thus capturing temporal dependencies inherent in time-series data. Reconstruction methods, including auto-encoders and diffusion models, aim to recover original data from corrupted or incomplete observations, facilitating the generation of features that encapsulate the essential characteristics of the dataset. By approximating the data distribution, generative-based methods significantly enhance the quality of features used in representation learning, allowing models to better understand the data’s complexities. Prominent examples of generative-based methods include reconstruction-oriented approaches such as SimMTM [30], TimeNet [31], PT-LSTM-SAE [32], and Autowarp [33], each of which offers unique mechanisms for effectively modeling and reconstructing temporal data.

In contrast, contrastive-based methods prioritize the relationships between different data instances. By forming pairs of similar (positive) and dissimilar (negative) data points, these methods train models to maximize the distance between negative pairs while minimizing the distance between positive pairs in the learned representation space. This approach effectively cultivates a nuanced understanding of data variation, enabling the model to discern subtle differences that are critical for effective classification and other tasks. The contrastive paradigm has gained traction in various domains, particularly in image and time-series analysis, due to its ability to learn robust, discriminative features essential for subsequent decision-making processes.

Adversarial methods introduce a competitive dynamic through the use of two neural networks: the generator and the discriminator. The generator aims to produce realistic data instances, while the discriminator strives to distinguish between genuine and synthesized data. This adversarial process fosters the development of highly effective representations by continuously challenging the model to improve its output, thereby enhancing the quality of the learned features. By incorporating adversarial training, models can achieve greater resilience and adaptability in diverse applications, including data augmentation and anomaly detection.

Collectively, these self-supervised learning strategies not only bolster the efficacy of representation learning but also pave the way for advancements in a wide array of machine learning tasks. By reducing dependence on labeled data and enhancing the richness of learned features, self-supervised learning holds considerable promise for addressing real-world challenges, particularly in fields where data annotation is labor-intensive or impractical. As we continue to explore and refine these methodologies, their impact on the future of machine learning and data analysis will undoubtedly become increasingly profound.

### 2.2. Contrastive Learning in Time Series Representation

Contrastive learning has emerged as an essential approach within self-supervised learning. It is designed to extract meaningful data representations by contrasting positive and negative sample pairs. At the heart of this method lies the proposal and selection of positive and negative samples, which are critical in guiding the model to learn distinctive features that capture the underlying structure of the data. By maximizing the similarity between positive pairs while minimizing it for negative pairs, contrastive learning enables a model to form well-differentiated representations, enhancing its capacity to recognize complex patterns.

Various frameworks have been proposed to implement contrastive learning, each tailored to different data structures and sampling aspects. For example, sampling-based approaches like Temporal Neighborhood Coding generate positive and negative samples by selecting instances based on their temporal proximity [34], effectively utilizing the natural sequence structure of time-series data. This ensures that temporally adjacent points, which often hold meaningful correlations, are reinforced in the learning process, while distant points serve as contrasts.

Furthermore, methods such as SimCLR [35], TS-TCC [26], and BTSF [36] represent advancements in time-series representation learning by employing effective data augmentation techniques. Through augmentation, these methods create multiple views of the same data, allowing the model to explore different perspectives and deepen its understanding of temporal dependencies. Contrastive learning using fixed masking techniques such as random cropping, jittering, and scaling allows for the extraction of classifiable features by learning commonalities across different views of the signal. However, these methods often struggle to capture the correlated characteristics of time-series data, particularly when the features are closely tied to temporal dependencies. The masking augmentation method proposed in this study addresses this limitation by adopting a learnable approach that reconstructs missing segments based on the underlying patterns within the signal, offering an innovative means to capture distinctive features more effectively.

### 2.3. Reconstruction-Based Method in Time Series Representation

Reconstruction-based methods aim to capture and learn the data’s underlying distribution to enable accurate reconstruction. These methods generally rely on an encoder–decoder architecture: the encoder transforms the input signal into a latent vector optimized for reconstruction, capturing the essential characteristics of the data in a compressed form, and the decoder then leverages this latent vector to reconstruct the original data as closely as possible.

In a typical reconstruction-based approach, the encoder and decoder are trained jointly, facilitating a learning process that reinforces the encoder’s ability to filter meaningful features for reconstruction. Once trained, the encoder primarily plays the role of a feature extractor, producing a robust representation, denoted as z, which serves as an input for downstream tasks such as classification, anomaly detection, and forecasting [37,38]. This transferability of the encoder’s output to downstream tasks underlines its versatility and effectiveness as a foundational component in representation learning.

In our study, we adopt a similar reconstruction-based strategy, incorporating a feature encoder specifically designed to support downstream tasks. By focusing on the encoder’s ability to capture relevant temporal features, we optimize its utility as a feature extractor module to learn the augmented signal’s distribution, enhancing its capacity to produce robust representations for diverse applications in time-series analysis.

## 3. Methodology

In this section, we introduce a structured framework designed to advance the learning of time-series representations through a sequence of carefully defined stages. Our approach moves away from traditional image-based augmentations, which often struggle to effectively capture the unique temporal dependencies and continuity inherent in time-series data. Instead, we integrate a learnable masking augmentation with a pre-training task that enables the model to extract both global and local features, which is crucial for capturing the rich structure of time-series data. Our key model features a two-stage framework. In the pre-training stage, an encoder–decoder structure learns features focused on reconstruction, which are aggregated via a feature aggregation module for downstream tasks. In the training stage, a multi-channel pyramid structure refines features at different channel scales. These features are refined through a feature refinement network using channel-wise concatenation and convolutional operations, enhancing their representational power for effective downstream performance.

During feature encoder training for downstream tasks, the use of an encoder–decoder architecture alone may interfere with contrastive loss optimization, leading to suboptimal representations. To address this, we adopt a multi-stage learning approach, initially training the feature encoder to reconstruct the augmented time-series inputs, thereby enhancing its capacity to extract discriminative features relevant to time-series continuity and dynamics. Once the encoder has been trained, we refine the extracted latent vectors to prepare them for contrastive learning. This ensures the model can effectively differentiate between relevant positive and negative pairs in the time-series data. A fine-tuning layer incorporating labeled data is trained in the final stage to achieve optimal classification performance.

**Figure 3:**
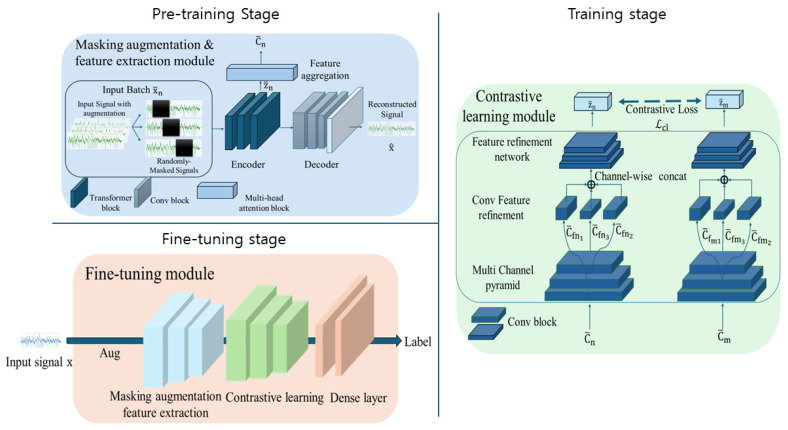
Overview of our proposed framework. The proposed framework consists of three distinct stages. In the pre-training stage, the framework pairs augmented signals with original signals, utilizing reconstruction to capture patterns within the time-series data. This stage leverages an encoder–decoder structure. During the training stage, extracted features are passed through a multi-channel pyramid and a feature-refinement network designed to enhance the latent vector’s view of different aspects. Finally, in the fine-tuning stage, the two models are integrated, and the dense layer is employed to train on both the original signals and their associated labels, refining the learned representation.

This methodology not only enhances representation quality for downstream tasks but also holds the potential for performance improvements when applied to other modules. Figure 3 provides an overview of the framework, illustrating each process stage. Detailed descriptions and analyses of these methods are provided in the following subsections.

### 3.1. Pre-Training Stage

The pre-stage consists of three components: a feature encoder, a decoder, and a feature-aggregation model. For the encoder, we incorporate transformers, which excel at capturing long-range dependencies and complex relationships, as demonstrated in natural language processing [39]. This allows the model to effectively map intricate patterns within time-series data. On the other hand, the decoder reconstructs the signal by processing the encoded features through a series of convolution blocks, followed by MLP layers. Input batches are constructed by pairing signals with random masks and their corresponding original signals. This creates a training dataset that encourages the model to learn meaningful correlations between the signal and augmented signals. This structure can be expressed mathematically as follows.

(1) $$\begin{array}{c} \bar{X_{n}}=Aug\left\{X\right\}\cup Mask\left\{X\right\} \end{array}$$

In Equation (1), ${\bar{X}}_{n}$ represents the set comprising the original signal *X* with time-based augmentation and its masked augmentation version. In it, the bar represents augmentation. In contrastive learning, augmentations that provide distinct data views are essential. Inspired by TS-TCC, we applied a mix of strong augmentations—such as permutation and jitter as well as a weaker augmentation strategy, scale-jitter, applied randomly in the time augmentation process. These augmentations are denoted by *n*, *m*, respectively.

(2) $$\begin{array}{c} \bar{Z_{n}}=\sum_{k}TrEncoder\left(\bar{X_{n}}\right) \end{array}$$

In the encoder stage, features are extracted by sequentially stacking transformers on the input batch. In Equation (2), Trencoder denotes the encoder part of the transformer, *k* indicates the sequence order of the extracted layers and represents the set of extracted features. We adopt a k value of 3 to ensure the following results:

(3) $$\bar{C_{a}}=softmax\left(\frac{QK^{T}}{\sqrt{D}}\bar{Z_{n}}\right)$$

(4) $$\begin{array}{c} \bar{C_{n}}=\bar{Z_{n}}*\bar{C_{a}} \end{array}$$

Equations (3) and (4) present the application of multi-head attention by concatenating features extracted from the transformer encoder to obtain the final middle-feature representation. To manage the increased feature dimensionality, two convolution layers were added for dimensionality reduction. Here, $\bar{C_{a}}$ represents the weights derived from the self-attention of $\bar{Z_{n}}$, while $\bar{C_{n}}$ is the resulting augmented feature set, and Q, K, D is Query, Key, and feature dimension of $\bar{Z_{n}}$.

### 3.2. Training Stage

The training stage involves a contrastive learning process in which two features obtained from distinct augmentations are compared to learn shared representations. Here, *n* and *m* denote strong and weak augmentations, respectively. Strong augmentation includes techniques such as permutation, jitter, shift, and scaling, which are randomly applied to the signal before entering the pre-training stage.

(5) $$\begin{array}{c} \bar{C_{fn1}}=Conv1\left(\bar{C_{n}}\right) \end{array}$$

(6) $$\begin{array}{c} \bar{C_{fn2}}=Conv2\left(\bar{C_{f}n1}\right) \end{array}$$

(7) $$\begin{array}{c} \bar{C_{fn3}}=Conv3\left(\bar{C_{f}n2}\right) \end{array}$$

Equations (5)–(7) depict the inputs and outputs of the sequential multi-channel pyramid, where each feature output progressively decreases in dimensionality from a channel perspective. This structured reduction allows for more refined feature extraction and enhances the model’s ability to capture essential patterns within the data, ultimately contributing to improved performance in downstream tasks [40]. Through convolution feature refinement, information from distinct layers is extracted via convolution blocks, leading to a progressive refinement of the channel representations.

(8) $$\begin{array}{c} \bar{z_{n}}=SeqConv\left(\bar{C_{f}n1}⊕\bar{C_{f}n2}⊕\bar{C_{f}n3}\right) \end{array}$$

Equation (8) represents the formalized process used in the training stage to derive the final latent vector for learning contrastive views. The SeqConv layer serves as a convolution layer for filtering channels of the concatenated features channel-wise, ultimately extracting $\bar{z_{n}}$ with a final dimensionality of 64 channels. During the training stage, the model utilized in the preceding pre-training phase for feature extraction remains frozen and is not subject to further training. Instead, the feature refinement network is trained to facilitate the contrastive learning process.

### 3.3. Fine-Tuning Stage

In the fine-tuning stage, the model is configured to accept the time-augmented signals as input, thereby establishing a sequential connection between each component utilized in the earlier stages. This integration culminates in adding a final dense layer, which enables a supervised learning framework. By incorporating this layer, the model can directly leverage the labels, ensuring that they play a role in guiding the training process and refining the learned representations for improved performance on the task at hand [41].

### 3.4. Loss

The loss function proposed in this study is categorized into two training steps: one for the pre-training stage and the other for the training stage. The losses defined in Equations (9)–(13) are designed to effectively capture the distribution of signals during the pre-training stage, ensuring the model learns the underlying structure of the data. In contrast, Equations (14) and (15) represent the loss functions used during the training stage to facilitate learning from different views of the data. The objective of the pre-training stage is to effectively comprehend and reconstruct the masked portions of the original signal; hence, reconstruction loss is utilized. Additionally, we adopt the paired constraint loss utilized in simMTM [30], incorporating a constraint loss to ensure that the elements within the masked signals achieve representations that closely resemble those of the original time series. Additionally, in the context of contrastive learning, we employed contrastive loss to facilitate the learning of shared characteristics among the feature vectors. An explanation of each loss is provided in the following subsections.

#### 3.4.1. Reconstruction Loss


(9) $$\begin{array}{c} loss_{re}=\sum_{i=1}^{N}{∥x_{i}-{\hat{x}}_{i}∥}_{2} \end{array}$$

Examining each term reveals that it consists of the $l_{2}$ norm of the time-augmented original signal $x_{i}$ and the reconstructed signal ${\hat{x}}_{i}$.

#### 3.4.2. Pair-Wised Loss

The pairwise loss function treats the original signal and the masked version of the original signal as a positive pair while considering the masked signals from different sources as negative pairs. By applying Kullback–Leibler divergence, this loss function facilitates contrasting between pairs, thereby enabling the acquisition of representations that are closely aligned.

(10) $$\begin{array}{c} \log_{–}\mathrm{softmax}\left(z_{i}\right)=\log\left(\mathrm{softmax}(z_{i})\right)=z_{i}-\log\left(\sum_{j}e^{z_{j}}\right) \end{array}$$

(11) $$\begin{array}{c} D_{KL}\left(P||Q\right)=\sum_{i}P\left(i\right)\log\left(\frac{P(i)}{Q(i)}\right) \end{array}$$

(12) $$\begin{array}{c} {\mathrm{loss}}_{\mathrm{pair}}=-D_{KL}(\log_{–}\mathrm{softmax}\left(z_{l}\right)||\log_{–}\mathrm{softmax}\left(z_{i}\right)) \end{array}$$

The variable $z_{i}$ represents the logits that encompass both positive and negative pairs, and the variable $z_{l}$ represents the logits that encompass only positive pairs. By comparing the normalized results obtained from Equation (10) with the distribution of the original signal in the context of Kullback–Leibler divergence in Equation (11), we can effectively learn the masking augmentation outcomes that exhibit distributions in close proximity from a pairwise perspective.

(13) $$\begin{array}{c} \mathrm{loss}={\mathrm{loss}}_{\mathrm{re}}+\gamma \cdot {\mathrm{loss}}_{\mathrm{pair}} \end{array}$$

Thus, the overall loss function consists of a weighted sum of the reconstruction loss and the pairwise loss. In this study, we adjusted the magnitude of each term during each training stage to ensure consistency between the two terms.

#### 3.4.3. Contrastive Loss

At the training stage, we employ the TS-TCC approach to capture similarity patterns in the time-series representations effectively [26] for the contrastive loss function. This approach leverages contrastive learning principles, where positive and negative pairs are contrasted to refine the feature space for meaningful representations. Specifically, positive pairs consist of the augmented signal and its corresponding original signal, while negative pairs are formed with unrelated signals.

(14) $$\begin{array}{c} L_{cl}=-\sum_{a=1}^{2N*(k+1)}\sum_{b=1}^{2N*(k+1)}\log\frac{I_{\left[n\neq m\right]}I_{\left[a=b\right]}\exp\left(sim\left({\bar{z}}_{n}^{a},{\bar{z}}_{m}^{b}\right)\right))}{\sum_{t=1}^{2N*(k+1)}I_{\left[a\neq t\right]}\exp\left(sim\left({\bar{z}}_{n}^{a},{\bar{z}}_{m}^{t}\right)\right)} \end{array}$$

(15) $$\begin{array}{c} \mathrm{sim}\left(u,v\right)=\frac{u^{T}v}{∥u∥∥v∥} \end{array}$$

The contrastive loss function, as shown in Equations (14) and (15), computes the similarity between these pairs, pushing positive pairs closer and negative pairs farther in the latent space. When the number of original signals is *N*, the total number of signals, including the original and *K* masking-augmented versions, is 2N(K+1). For the *a*-th sample, the similarity between ${\bar{z}}_{n}^{a}$ and ${\bar{z}}_{m}^{a}$ is measured using a similarity function of Equation (15), which indicates different fixed augmentation in a similar manner to the pairwise loss calculation. I represents indicator function to select pairs in the dataset. This setup allows the model to focus on relevant temporal patterns, ultimately enhancing the robustness and generalization of learned representations across different time-series data.

## 4. Experiment

The experiments were carried out using multiple time-series datasets, including HAR [27], Sleep-EDF [28], and Epilepsy [29], to comprehensively evaluate the proposed method across various domains. The HAR dataset was chosen for its representation of human activity recognition tasks, providing insights into how the model performs with sensor-based movement data. The Sleep-EDF dataset was used to assess the model’s ability to handle physiological signals related to sleep stages, which involve complex temporal dependencies. Finally, the Epilepsy dataset was employed to test the robustness of the model in detecting neurological patterns. This diverse set of datasets enabled us to demonstrate the effectiveness and adaptability of the proposed approach across different types of time-series data.

### 4.1. Datasets

#### 4.1.1. Sleep-EDF

The Sleep-EDF dataset consists of 197 full-night polysomnographic recordings, which include signals such as EEG (electroencephalogram), EOG (electrooculogram), and chin EMG (electromyogram). These recordings provide a comprehensive view of physiological activities during sleep, enabling detailed analysis of different sleep stages. In this study, we specifically used EEG data to classify sleep stages, as EEG is widely recognized for its effectiveness in capturing neural activity and identifying distinct sleep phases. We used a single EEG channel at a sampling rate of 100 Hz, following previous studies [26]. Our approach aims to leverage the rich temporal and spectral features inherent in brain activity to achieve accurate sleep stage classification.

#### 4.1.2. HAR

The HAR dataset [27] consists of inertial sensor data collected from participants holding smartphones, which capture six distinct types of human activities. With a sampling rate 50 Hz, we stacked three components, Accelerometer, Gyroscope, and Gravity, with a total of 9 channels of x, y, and z elements. These activities include walking, sitting, standing, and other common motions, providing a comprehensive representation of everyday behaviors. Data were gathered from 30 subjects, ensuring diversity in the dataset and enabling the evaluation of the model across varying individual characteristics. The dataset’s rich collection of accelerometer and gyroscope readings makes it well suited for testing models designed for human activity recognition, providing both temporal complexity and a wide range of motion patterns.

#### 4.1.3. Epilepsy

The Epileptic Seizure Recognition dataset, much like the Sleep-EDF dataset, contains EEG recordings capturing brain activity from 500 individual instances. For a fair comparison, we followed the procedure of dataset division in [26] by combining all non-seizure labels into a single category, effectively focusing on a binary classification between seizure and non-seizure states to compare our methods between contrastive learning methods. The dataset originally included five distinct labels representing various brain states, including seizure activity.

### 4.2. Implementation Details

#### 4.2.1. Augmentation

For augmentation, to create contrastive views from the input, we applied scaling, permutation, and scaling with jitter, each with a 50% probability of being applied concurrently. The parameters for each augmentation were as follows: scaling factors ranged from 0.5 to 1.5; jitter was applied with a sigma scale from 0 to 0.8; and permutation was performed by dividing the data into five segments and randomly selecting split points. Additionally, masking augmentation was implemented with a masking ratio of 0.25.

#### 4.2.2. Parameters

In terms of the model architecture, the encoder transformer used for pre-training consisted of three blocks with a dimension of 128, which was also the dimension of the decoder. The dimensions were set to 256, 128, and 64 for the convolutional blocks used in the training stage. Finally, in the second layer, 64 channels were selected for output. A batch size of 128 was used, and each epoch was set to 30 s for the Sleep-EDF dataset.

#### 4.2.3. Environment

The experiments were conducted in a PyTorch 1.7.1 environment, utilizing an NVIDIA Geforce Single RTX 3090 GPU, 64 GB RAM, and an Intel Core i9 CPU.

### 4.3. Comparative Result

Table 1 provides a comprehensive comparison between the proposed method and existing approaches across three prominent datasets: Sleep-EDF, UCI HAR, and Epilepsy. The Sleep-EDF dataset is divided into two subsets based on the number of subjects and the number of folds: SleepEDF-20 and SleepEDF-78. For SleepEDF-78, our method achieved an accuracy improvement of 2% and a gain of approximately 2.6 in the MF1 score compared to existing approaches. For the SleepEDF-20 dataset, the proposed method demonstrated superior performance compared to TS-TCC, which our approach was inspired by and which used the same augmentation strategies as this study. Specifically, our model achieved a 2.55% improvement in accuracy and an approximately 1.96-point increase in the MF1 score, highlighting significant gains in classification performance.

In the UCI HAR experiments, our approach exhibited a substantial performance improvement over the previously applied masked reconstruction method, showing a significant 12.23-point increase in the MF1 score. Furthermore, compared to TS-TCC, our method demonstrated notable gains, with a 3.74-point increase in MF1 score and a 3.89% increase in accuracy. It is worth noting that accuracy values were not publicly available for some methods applied to the UCI HAR dataset and are therefore marked as None in the table.

For the Epilepsy dataset, a binary classification was conducted, and comparisons were made against four methods previously disclosed in TS-TCC. The proposed method achieved results comparable to the baseline without significant differences, indicating that our approach is on par with the current methods for this dataset. Overall, the results presented in Table 1 demonstrate the effectiveness and adaptability of our proposed method across a diverse set of datasets, illustrating its potential for improving classification performance in different time-series domains.

### 4.4. Ablation Studies

Table 2 provides a detailed overview of the classification results for the largest dataset in this study, SleepEDF-78. The metrics include precision (PR), recall (RE), and the F1 score (F1), each offering insight into the model’s performance for different classes. The vertical axis of the table represents the actual labels—Wake (W), N1, N2, N3, and REM (R)—while the horizontal axis denotes the predicted classes. This confusion matrix allows for a thorough analysis of the prediction accuracy for each label, offering a comprehensive understanding of how well the model distinguishes between different sleep stages. Additionally, it illustrates any misclassifications and the distribution of errors, providing valuable insights for further optimization of the model.

Table 3 provides the accuracy and MF1 scores corresponding to different masking ratios, highlighting one of the contributions of this study. The results indicate that the highest performance was achieved with a masking ratio of 0.25. Additionally, when the masking ratio was set to zero—meaning that only time augmentation was applied without masking—the performance was notably lower compared to other ratios. The performance difference of the model according to the masking ratio can be attributed to variations in feature reconstruction capabilities. With a higher masking ratio, the model must restore more of the original signal’s features, leading to a reliance on global features for reconstruction. Conversely, with a lower masking ratio, the model focuses more on local features. This results in a trade-off between global and local feature utilization. In our experiments, we determined an optimal masking ratio of 0.25. This finding underscores the effectiveness and importance of the masking technique in enhancing the model performance.

**Figure 4:**
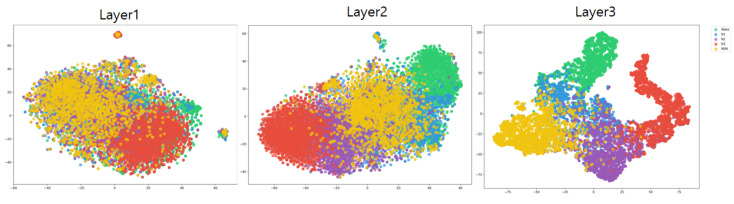
T-SNE visualization of the feature refinement model across different layers.

Figure 4 presents the t-SNE visualization of the data as they pass through the feature-refinement network during the training stage. The visualization highlights how feature aggregation becomes progressively more organized and distinct as the data move through each successive network layer. This demonstrates that the feature refinement network effectively enhances the representation of the data, ultimately leading to improved structure and separability in the latent space.

## 5. Discussion

The integration of masking augmentation and contrastive learning within a unified framework has proven effective in extracting meaningful representations from complex time-series data. One of the key insights derived from this study is the importance of strategically masking data segments, allowing the model to focus on both the continuity and critical transitions inherent in temporal sequences. Compared to traditional methods which adopted only fixed augmentation, our learnable masking augmentation optimized the balance between data reconstruction and feature refinement, thus effectively bridging the gap between handcrafted augmentations and fully learnable, data-driven processes. Nonetheless, challenges remain for the efficient integration of the model. Automating the parameters of the masking network and applying quantization for model compression are essential steps for practical deployment. Additionally, incorporating the characteristics of larger and more diverse datasets could enable the development of a network that better captures time-variant features, paving the way for further improvements. Future work could also explore the integration of other forms of self-supervised learning, such as adversarial training, to complement the current approach and further enrich the quality of the learned representations.

## 6. Conclusions

In this study, we presented a novel framework that leverages a learnable masking augmentation strategy within a contrastive learning setup for time-series representation learning. By introducing learnable masking and reconstruction techniques, the model effectively captured both global and local temporal dependencies, enhancing the quality of feature extraction for downstream tasks. Experimental evaluations on multiple benchmark datasets, including HAR, Sleep-EDF, and Epilepsy, demonstrated that our approach not only outperforms existing methods but also shows robustness and generalizability across various domains of time-series data. These results highlight the potential of the proposed framework in advancing time-series analysis by creating more discriminative and context-aware representations, ultimately improving the performance of time-series models. 

## Figures and Tables

**Table 1:** Performance overview of the classification performance achieved on the 4 different datasets. It includes the metrics of accuracy and MF1 score, allowing for a comparison of different methods evaluated in the study. The best results are displayed in bold.

Dataset	Method	ACC (%)	MF1
SleepEDF-78	SLEEPEEG [42]	80	73.6
	DEEPSLEEP [43]	80.8	74.2
	U-TIME [44]	81.3	76.3
	transformer [45]	81.4	74.3
	1-MAXCNN [46]	81.9	73.8
	SEQSLEEP [47]	82.6	76.4
	**Ours**	**84.6**	**79.0**
SleepEDF-20	SSL-ECG [48]	74.58	65.44
	SIMCLR [35]	78.91	68.6
	CPC [49]	82.82	73.94
	TS-TCC [26]	83	73.57
	**Ours**	**85.55**	**75.53**
UCI-HAR	SSL-ECG [48]	65.34	63.75
	SIMCLR [35]	80.97	80.19
	Multi-taskSSL [50]	None	89.81
	MaskedRe [51]	None	81.89
	ClusterCLHAR [52]	None	92.63
	DeepConvLSTM [53]	82.6	76.4
	CPC [49]	83.85	83.27
	TS-TCC [26]	90.37	90.38
	TNC [34]	92.03	None
	STF-CSL [54]	93.96	94.10
	**Ours**	**94.26**	**94.12**
Epilepsy	SSL-ECG [48]	93.72	89.15
	SIMCLR [35]	96.05	93.53
	CPC [49]	96.61	94.44
	TS-TCC [26]	**97.23**	95.54
	**Ours**	97.12	**96.25**

**Table 2:** The confusion matrix results and performance metrics for each label for the SleepEDF-78 dataset.

SleepEDF-78								
	W	N1	N2	N3	R	PR	RE	F1
W	63,893	3562	327	25	640	93.6	93.3	93.5
N1	3529	9914	6182	58	1839	55.8	46.1	50.5
N2	506	3071	61,406	1861	2288	84.2	88.8	86.5
N3	45	33	2822	10,125	14	83.8	77.7	80.6
R	279	1198	2157	12	22,189	82.3	85.9	84.0

**Table 3:** Classification results on the SleepEDF-78 dataset based on varying masking ratios.

SleepEDF-78		
Masking Ratio	ACC	MF1
0	83.5	76.9
0.1	83.9	77.5
0.25	84.6	79.0
0.3	84.3	78.8

## Data Availability

The corresponding acquired training and testing data are available through a public site.
